# Factors associated with acquiring exercise habits through health guidance for metabolic syndrome among middle-aged Japanese workers: A machine learning approach

**DOI:** 10.1016/j.pmedr.2024.102915

**Published:** 2024-10-19

**Authors:** Jiawei Wan, Kyohsuke Wakaba, Takeshi Onoue, Kazuyo Tsushita, Yoshio Nakata

**Affiliations:** a Graduate School of Comprehensive Human Sciences, University of Tsukuba, Tsukuba 305-8574, Japan; b Faculty of Human Life, Jumonji University, Niiza 352-8510, Japan; c Department of Endocrinology and Diabetes, Nagoya University Graduate School of Medicine, Nagoya 464-8601, Japan; d Faculty of Nutrition, Kagawa Nutrition University, Sakado 350-0288, Japan; e Institute of Health and Sport Sciences, University of Tsukuba, Tsukuba 305-8574, Japan

**Keywords:** Exercise habit, Metabolic syndrome, Health guidance, National database, Machine learning

## Abstract

**Objective:**

Physical inactivity increases the risk of mortality and chronic morbidity. Therefore, it is crucial to establish strategies to encourage individuals to increase their physical activity and develop exercise habits. The objective of this study was to explore factors associated with acquiring exercise habits using machine learning algorithms.

**Methods:**

The analyzed dataset was obtained from the Specific Health Guidance for metabolic syndrome systematically implemented by the Japanese Ministry of Health, Labor, and Welfare. We selected target individuals for health guidance without exercise habits in 2017 and assessed whether the participants acquired exercise habits through health guidance in 2018. We applied ten machine learning algorithms to build prediction models for acquiring exercise habits.

**Results:**

This study included 16,471 middle-aged Japanese workers (age, 49.5 ± 6.2 years). Among the machine learning algorithms, the Boosted Generalized Linear Model was the best for predicting the acquisition of exercise habits based on the receiver operating characteristic curve on the test set (ROC-AUC_test_, 0.68). According to the analyses, the following factors were associated with the acquisition of exercise habits: being in the maintenance or action stage of changing exercise and eating behaviors based on the transtheoretical model; regular physical activity or walking; normal high-density lipoprotein cholesterol; and high alcohol consumption.

**Conclusions:**

Our findings can be used to establish an efficient strategy for encouraging individuals to acquire exercise habits through Specific Health Guidance or other health guidance. However, the lower ROC-AUC_test_ suggests that additional variables are necessary to enhance the prediction model.

## Highlights

• Machine learning explored primary factors influencing exercise habit acquisition.
• Data of high clinical value were sourced from 22 health insurers, Japan.
• Transtheoretical model/regular physical activity affect exercise habit acquisition.
• Main factors influencing and predicting exercise habit acquisition were identified.
• Additional variables are necessary to enhance the prediction model.

## 1 Introduction

Physical inactivity is known to increase the risk of mortality and morbidity of chronic diseases, such as type two diabetes, cerebrovascular disease, and heart disease. The World Health Organization (WHO) reported that physical inactivity is the fourth leading risk factor for mortality after hypertension, smoking, and hyperglycemia (WHO, 2020). A 32-year cohort study including approximately 100,000 adults reported that regular exercise can substantially reduce the risk of mortality (Veronese et al., 2016). Therefore, it is necessary to establish strategies to encourage individuals to increase their physical activity and acquire exercise habits.

In 2008, the Japanese Ministry of Health, Labor, and Welfare (MHLW) implemented the revolutionary system of Specific Health Checkups (SHC) and Specific Health Guidance (SHG) for individuals with metabolic syndrome (MetS) and those at presumptive risk (pre-MetS) (Mizushima and Tsushita, 2011). SHC is a health assessment focused on MetS in Japanese individuals aged 40–74 years. SHG is a lifestyle intervention program (i.e., goal setting, physical activity, and diet) for individuals with MetS or pre-MetS performed by healthcare professionals. The SHG can be divided into intensive health guidance (IHG) and motivational health guidance (MHG). IHG provides ongoing follow-up and initial counseling to individuals diagnosed with MetS. MHG comprises a single health guidance session for individuals diagnosed with MetS or pre-MetS.

While both IHG and MHG were found to statistically improve factors related to MetS, the level of improvement may not be clinically meaningful (Matsushita et al., 2017, Muramoto et al., 2014, Tsushita et al., 2018). Given that regular exercise boosts the effect of IHG and MHG (Nakashita et al., 2013) and maintains the effect on weight loss (Kang et al., 2022), it is critical to focus on factors that influence the acquisition of exercise habits. Although a cross-sectional study suggested that physical activity levels may be associated with lifestyle factors among Japanese adults (Sumimoto et al., 2021), the longitudinal association between the acquisition of exercise habits and lifestyle factors remains unclear. Hence, to improve the effectiveness of MHG, it is essential to understand the factors that influence the acquisition of exercise habits.

Recently, machine learning approaches have been proposed as valuable tools for identifying the behavioral, social, and environmental factors that impact health-related behaviors. During data analysis using machine learning, a computer automatically learns the data to identify the background and predict outcomes (Barrett et al., 2013). Despite the advantages of machine learning approaches, limited information is available on lifestyle factors associated with acquiring exercise habits among Japanese individuals. Therefore, in this study, we aimed to explore factors related to the acquisition of exercise habits using a machine learning approach by employing large-scale accumulated data from Japanese workers who underwent SHC and MHG as a secondary source.

## 2 Methods

### 2.1 Study design

This was a longitudinal observational study conducted using large-scale accumulated data by SHC and MHG. Fig. A.1 presents the study flow.

### 2.2 Data source and study population

We obtained and analyzed a database from 22 health insurers, including the Japan Health Insurance Association, Mutual Aid Associations for Municipal Personnel, and National Health Insurance Associations in Japan. This database contains data on Japanese workers who underwent SHC for two consecutive years (2017 and 2018).

A total of 47,808 individuals aged 40–64 years (36,038 males and 11,770 females) who underwent SHC for two consecutive years and had completed MHG were included. The SHC and MHG were conducted as part of a public undertaking; thus, the present study was not a clinical trial.

Baseline and follow-up data were collected in 2017 and 2018, respectively. Individuals with exercise habits in 2017 (24,930 individuals) and those with missing variables at the time of the baseline survey (8,459 individuals) were excluded from our analyses. A total of 16,471 participants (age, 49.5 ± 6.2 years) were included in the analysis.

The study followed the Strengthening the Reporting of Observational Studies in Epidemiology reporting guidelines for cohort studies, was conducted in accordance with the Declaration of Helsinki, and was approved by the local Ethics Committee of Kagawa Nutrition University on October 21, 2020 (No. 304) and the Ethics Committee of the University of Tsukuba on December 23, 2020 (Tai 020-135). Given the retrospective and anonymized nature of the study, the requirement for written informed consent from the participants was waived.

### 2.3 SHC and MHG

The details regarding the SHC and MHG have been published elsewhere (Tsushita et al., 2018, Ministry of Health, Labor, and Welfare, 2024). Briefly, the SHC is an annual health checkup for individuals aged 40–74 years and includes clinical tests, questionnaires, and physical examinations to assess risk factors for MetS. Measurements of the following parameters are part of the clinical tests and physical examination: waist circumference, body weight, body mass index (BMI), systolic blood pressure (SBP), diastolic blood pressure (DBP), aspartate transaminase (AST), alanine transaminase (ALT), gamma-glutamyl transpeptidase (γ-GTP), hemoglobin A1c (HbA1c), serum triglycerides, high-density lipoprotein cholesterol (HDL-C), and low-density lipoprotein cholesterol (LDL-C). The questionnaire assessed whether the participants were smokers and whether they took medication for diabetes, hypertension, or dyslipidemia. Individuals undergoing specific health checkups were screened based on obesity-related indicators (waist circumference and BMI) and additional metabolic risk factors. Based on these results, individuals determined to be at high risk were eligible for MHG.

### 2.4 Candidate predictors and outcomes

We extracted 33 candidate predictors for machine learning from the SHC baseline data, including anthropometric measurements, metabolic parameters, and self-administered questionnaires. Anthropometric measurements included age, body weight, waist circumference, BMI, SBP, and DBP. The metabolic parameters included baseline SBP, DBP, AST, ALT, γ-GTP, triglycerides, LDL-C, HDL-C, and HbA1c. Results were categorized as normal and abnormal according to the SHG guidelines. Predictors categorized according to the SHG guidelines were added to the STRING “ctg,” which means “categorized.” Measure data were categorized into four seasons (Season One: 4/1/2017–5/31/2017, Season Two: 6/1/2017–8/31/2017, Season Three: 9/1/2017–11/30/2017, Season Four: 12/1/2017–3/31/2018). Age was categorized as >55 years and ≤55 years, and the string “55” indicates “>55 years of age.”

The self-administered questionnaire included questions on exercise habits, anemia, smoking habits, body weight gain of ≥10 kg from the age of 20 years, regular physical activity or walking at least one hour per day, walking speed, body weight changes of more than three kilograms in the past year, eating speed, late dinner, eating snacks after dinner, skipping breakfast, daily alcohol consumption of ≥60 g, sleep quality, stage of changes in lifestyle behavior (i.e., exercise and eating behavior) based on the transtheoretical model (TTM), and willingness to receive SHG. Table A.1 summarizes the definitions of variables derived from the questionnaire survey.

The outcome variable was a binary measure of exercise habit acquisition. Exercise habits were defined as participation in any physical activity for at least 30 min/time and two days/week that caused light sweating in the past 12 months. Exercise habits were assessed using a self-administered questionnaire.

Numerical predictors were centered and scaled using step_center and step_scale functions. Categorical predictors were dummied using step_dummy function from recipes package version 1.0.8 (Kuhn et al., 2024).

### 2.5 Feature selection

Feature selection was performed using Least Absolute Shrinkage and Selection Operator (LASSO) regression with glmnet package (Tay et al., 2023). To determine the best regularization rate for the LASSO regression, cv.glmnet function with 10-fold cross-validation was used. Lambda with a minimum mean cross-validated error was used as the best lambda value.

### 2.6 Variance inflation factor (VIF)

We performed a VIF test to detect multicollinearity using the VIF function from car package version 3.1.2 (Fox and Weisberg, 2019). The presence of high multicollinearity was considered if the VIF of the variable was greater than five.

### 2.7 Machine development

To predict the acquisition of exercise habits, we employed 10 algorithms (Logistic Regression, Classification and Regression Trees, LASSO and Elastic-Net Regularized Generalized Linear Model, Penalized Multinomial Regression, Random Forest, Support Vector Machines with Linear Kernel, Partial Least Squares, Boosted Classification Tree, Extreme Gradient Boosting [xgboost], Boosted Generalized Linear Model [BGLM]). The R packages, brief instructions, and versions of the 10 machine learning algorithms are summarized in Table A.2.

### 2.8 Model training

Model training was conducted using the train function from caret package version 6.0.94 (Kuhn, 2008). Exercise habits in 2018 were set as outcomes, and the remaining variables were imported as candidate predictors.

Owing to the imbalance in the ratio of positive and negative samples, we employed the synthetic minority oversampling technique to prevent oversampling (Chawla et al., 2002).

### 2.9 Model evaluation

The area under the receiver operating characteristic (ROC) curve (ROC-AUC) was used as a metric to evaluate the model. The equations for calculating the sensitivity, specificity, and accuracy of the models are shown in the Eqs. (1), (2), (3). While tuning the hyperparameters of the models and evaluating their performance, we selected the combination of hyperparameters that resulted in the highest ROC-AUC in the model.

(1) $$\mathrm{Sensitivity}=\frac{\text{True positives}}{\text{True positives}+\text{False negatives}}$$

(2) $$Specificity=\frac{\text{True negatives}}{\text{True negatives}+\text{False positives}}$$

(3) $$Accuracy=\frac{\text{True positives}+\text{True negatives}}{\text{True positives}+\text{True negatives}+\text{False positives}+\text{False negatives}}$$

### 2.10 Statistical analysis

P values <0.05 were considered statistically significant. Baseline characteristics are summarized as means and standard deviations for numerical variables and as frequencies and percentages for categorical variables. Unequal variance t-tests (Welch’s *t*-test) were used to assess the statistical significance of between-group differences, and the chi-square test was used to compare proportions using rstatix package version 0.7.2 (Kassambara, 2023). The VIF test was performed to confirm multicollinearity in models using the VIF function from car package version 3.1.2 (Fox and Weisberg, 2019). The best threshold, specificity, sensitivity, accuracy, and ROC-AUC of the model were calculated using pROC package version 1.18.4 (Robin et al., 2011). Variable importance was calculated using varImp function from caret package version 6.0.94 (Kuhn, 2008). Data analyses were conducted using R version 4.3.1 for Mac, an open-source statistical software package (R Foundation for Statistical Computing, Vienna, Austria).

## 3 Results

### 3.1 Characteristics of individuals

Table A.3 presents the characteristics of individuals included and excluded from the final analysis. There were significant differences in sex, season, age, body weight, waist circumference, BMI, SBP, DBP, γ-GTP, HDL-C, HbA1c, anemia, body weight gain of ≥10 kg from the age of 20 years, regular physical activity or walking at least one hour per day, faster walking speed than others, body weight gain of ≥10 kg from 2016 to 2017, eating speed, late dinner, skipping breakfast, daily alcohol consumption of ≥60 g, good sleep quality, stage of changes in lifestyle behavior based on the TTM, and willingness to receive SHG between individuals included (n = 16,802) and excluded in the final analysis (n = 9,960).

Table 1 presents the characteristics of individuals with and without exercise habits in 2018, one year after the baseline. Individuals with and without exercise habits exhibited significant differences in age, waist circumference, ALT, triglycerides, HbA1c, body weight gain of ≥10 kg from the age of 20 years, regular physical activity or walking at least one hour per day, walking speed, late dinner, skipping breakfast, daily alcohol consumption of ≥60 g, good sleep quality, and stage of changes in lifestyle behavior based on the TTM.

**Table 1:** Characteristics of Japanese workers at high risk for metabolic syndrome who received the Specific Health Guidance, by exercise adoption status one year after baseline, 2017–2018.

	**All**	**Individuals** **without Exercise Habits**	**Individuals** **with Exercise Habits**	P value*
	n = 16,471	n = 14,964	n = 1,507	
**Sex, n (%):**				0.12
**Female**	4,469 (27.1)	4,086 (27.3)	383 (25.4)	
**Male**	12,002 (72.9)	10,878 (72.7)	1,124 (74.6)	
**Season, n (%):**				0.10
**One**	4,709 (28.6)	4,280 (28.6)	429 (28.5)	
**Two**	4,685 (28.4)	4,234 (28.3)	451 (29.9)	
**Three**	3,881 (23.6)	3,562 (23.8)	319 (21.2)	
**Four**	3,196 (19.4)	2,888 (19.3)	308 (20.4)	
**Age, years**	49.5 (6.2)	49.5 (6.2)	50.0 (6.4)	<0.01
**Height, cm**	167.6 (8.6)	167.5 (8.6)	167.7 (8.4)	0.52
**Body weight, kg**	74.4 (9.3)	74.4 (9.3)	74.3 (9.2)	0.87
**Waist circumference, cm**	90.5 (6.7)	90.5 (6.7)	89.8 (6.5)	<0.01
**BMI, kg/m^2^**	26.5 (2.7)	26.5 (2.7)	26.4 (2.6)	0.33
**SBP, mmHg**	127.1 (14.8)	127.1 (14.8)	127.4 (14.6)	0.50
**DBP, mmHg**	80.0 (10.8)	80.0 (10.8)	80.1 (10.6)	0.63
**AST, U/L**	23.9 (10.8)	23.9 (10.8)	24.1 (10.6)	0.51
**ALT, U/L**	29.2 (20.0)	29.3 (20.2)	28.3 (18.3)	<0.05
**γ-GTP, U/L, U/L**	47.0 (46.7)	47.0 (46.5)	46.8 (48.6)	0.84
**Triglycerides, mg/dL**	127.9 (83.6)	128.3 (84.1)	123.8 (78.7)	<0.05
**HDL-C, mg/dL**	57.4 (13.8)	57.3 (13.8)	57.9 (13.7)	0.12
**LDL-C, mg/dL**	133.1 (29.8)	133.1 (29.9)	132.6 (29.5)	0.51
**HbA1c, %**	5.6 (0.4)	5.6 (0.4)	5.6 (0.5)	<0.01
**Anemia, n (%):**				0.18
**No**	15,379 (93.4)	13,959 (93.3)	1,420 (94.2)	
**Yes**	1,092 (6.6)	1,005 (6.7)	87 (5.8)	
**Smoker, n (%):**				0.83
**No**	15,859 (96.3)	14,406 (96.3)	1,453 (96.4)	
**Yes**	612 (3.7)	558 (3.7)	54 (3.6)	
**Body weight gain of** ≥**10 kg from the age of 20 years, n (%):**				<0.01
**No**	4,630 (28.1)	4,145 (27.7)	485 (32.2)	
**Yes**	11,841 (71.9)	10,819 (72.3)	1,022 (67.8)	
**Regular physical activity or walking at least one hour per day, n (%):**				<0.01
**No**	12,186 (74.0)	11,322 (75.7)	864 (57.3)	
**Yes**	4,285 (26.0)	3,642 (24.3)	643 (42.7)	
**Faster walking speed than others, n (%):**				<0.01
**No**	10,051 (61.0)	9,249 (61.8)	802 (53.2)	
**Yes**	6,420 (39.0)	5,715 (38.2)	705 (46.8)	
**Body weight changes of more than three kilograms from 2016 to 2017, n (%):**				0.28
**No**	10,522 (63.9)	9,579 (64.0)	943 (62.6)	
**Yes**	5,949 (36.1)	5,385 (36.0)	564 (37.4)	
**Eating speed, n (%):**				0.53
**Fast**	6,449 (39.2)	5,847 (39.1)	602 (39.9)	
**Normal or slow**	10,022 (60.8)	9,117 (60.9)	905 (60.1)	
**Late dinner time, n (%):**				<0.01
**No**	10,374 (63.0)	9,373 (62.6)	1,001 (66.4)	
**Yes**	6,097 (37.0)	5,591 (37.4)	506 (33.6)	
**Eating snacks after dinner, n (%):**				0.06
**No**	13,573 (82.4)	12,304 (82.2)	1,269 (84.2)	
**Yes**	2,898 (17.6)	2,660 (17.8)	238 (15.8)	
**Skipping breakfast, n (%):**				<0.01
**No**	13,759 (83.5)	12,453 (83.2)	1,306 (86.7)	
**Yes**	2,712 (16.5)	2,511 (16.8)	201 (13.3)	
**Daily alcohol consumption, n (%):**				<0.01
**Do not drink**	3,520 (21.4)	3,246 (21.7)	274 (18.2)	
**Less than 20 g**	5,910 (35.9)	5,370 (35.9)	540 (35.8)	
**20–40 g**	4,327 (26.3)	3,900 (26.1)	427 (28.3)	
**40–60 g**	1,970 (12.0)	1,759 (11.8)	211 (14.0)	
**More than 60 g**	744 (4.5)	689 (4.6)	55 (3.6)	
**Good sleep quality, n (%):**				<0.01
**No**	7,514 (45.6)	6,931 (46.3)	583 (38.7)	
**Yes**	8,957 (54.4)	8,033 (53.7)	924 (61.3)	
**Lifestyle behavior based on the TTM, n (%):**				<0.01
**Precontemplation**	3,117 (18.9)	2,927 (19.6)	190 (12.6)	
**Contemplation**	7,355 (44.7)	6,838 (45.7)	517 (34.3)	
**Preparation**	2,710 (16.5)	2,452 (16.4)	258 (17.1)	
**Action**	1,806 (11.0)	1,515 (10.1)	291 (19.3)	
**Maintenance**	1,483 (9.0)	1,232 (8.2)	251 (16.7)	
**Willingness to receive SHG, n (%):**				0.06
**No**	12,031 (73.0 %)	10,962 (73.3)	1,069 (70.9)	
**Yes**	4,440 (27.0 %)	4,002 (26.7)	438 (29.1)	

Note: Data are shown as mean (standard deviation) for continuous variables or n (%) for categorical variables. P values were generated using Welch’s *t*-test or chi-squared test.

Abbreviations: **BMI**, body mass index; **SBP**, systolic blood pressure; **DBP**, diastolic blood pressure; **AST**, aspartate transaminase; **ALT**, alanine transaminase; **γ-GTP**, gamma-glutamyl transpeptidase; **HDL-C**, high-density lipoprotein cholesterol; **LDL-C**, low-density lipoprotein cholesterol; **HbA1c**, hemoglobin A1c; **TTM**, transtheoretical model; **SHG**, Specific Health Guidance.

Definitions: **Exercise habits**, participation in any physical activity for at least 30 min/time and two days/week that caused light sweating in the past 12 months； **Season**, the dates SHG receipt were categorized into four seasons (Season One: 4/1/2017–5/31/2017, Season Two: 6/1/2017–8/31/2017, Season Three: 9/1/2017–11/30/2017, Season Four: 12/1/2017–3/31/2018); **Smoker**, had smoked a total of over 100 cigarettes or have smoked over a period of six months and have been smoking over the past month; **Regular physical activity or walking at least one hour per day**, whether walking or any equivalent amount of physical activity was performed for more than one hour a day in daily life; **Faster walking speed than others**, the participant thought his/her walking speed was faster than the speed of those of the same age and sex; **Eating speed**, whether the participant thought he/she ate faster than others; **Late dinner time**, eating supper two hours before bedtime more than three times a week; **Eating snacks after dinner**, eating snacks after supper more than three times a week; **Skipping breakfast**, skipping breakfast more than three times a week; **Good sleep quality**, whether the participant felt he/she slept well and sufficiently; **Willingness to receive SHG**, whether the participant wanted to use the opportunity of health instructions for improving his/her life habits.

### 3.2 Features selected by LASSO regression and VIFs

Table A.4 presents the results of the LASSO regression and variable names in the coding. All variables except height were selected using LASSO regression. The VIF of variables is shown in Table A.5. No variables with VIF greater than five were detected.

### 3.3 Comparison of the model performance

**Fig. 1:**
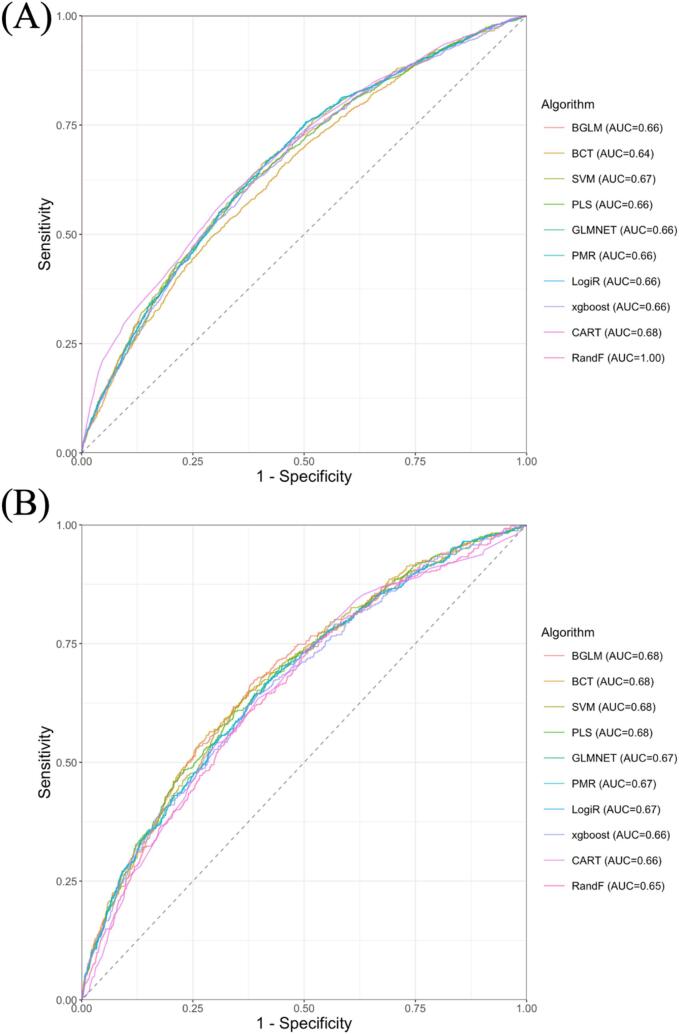
Receiver operating characteristic curves for 10 machine learning models* on the training (A) and test (B) sets involving Japanese workers at high risk for metabolic syndrome who received Specific Health Guidance, 2017–2018. Note: *Results are sorted in descending order by receiver operating characteristic curve values on the test set. Abbreviations: **AUC**, area under the curve; **BGLM**, boosted generalized linear model; **BCT**, boosted classification tree; **SVM**, support vector machine with linear kernel; **PLS**, partial least squares; **GLMNET**, lasso and elastic-net regularized generalized linear model; **PMR**, penalized multinomial regression; **LogiR**, logistic regression; **xgboost**, extreme gradient boosting; **CART**, classification and regression trees; **RandF**, random forest.

Table A.6 presents the results of the selected hyperparameters after repeated 10-fold cross-validation. Fig. 1 presents the ROC curves of the 10 models for the training and test sets. Overall, the BGLM (ROC-AUC on the test set, ROC-AUC_test_ = 0.68) had the best performance among the 10 models. The sensitivity, specificity, and accuracy were 0.62, 0.67, and 0.61, respectively. The ROC-AUC_test_, sensitivity, specificity, and accuracy of the 10 models and all models on the test set are summarized in Table 2.

**Table 2:** The ROC-AUC, sensitivity, specificity, and accuracy of 10 models on the test set involving Japanese workers at high risk for metabolic syndrome who received the Specific Health Guidance, 2017–2018.

**Algorithm**	**ROC-AUC_test_**	**Threshold**	**Accuracy**	**Sensitivity**	**Specificity**
**BGLM**	0.68	0.48	0.62	0.67	0.61
**BCT**	0.68	0.50	0.70	0.56	0.71
**SVM**	0.68	0.49	0.63	0.65	0.63
**PLS**	0.68	0.50	0.65	0.61	0.66
**GLMNET**	0.67	0.49	0.61	0.64	0.61
**PMR**	0.67	0.49	0.62	0.64	0.61
**LogiR**	0.67	0.49	0.62	0.64	0.61
**xgboost**	0.66	0.20	0.61	0.64	0.60
**CART**	0.66	0.16	0.61	0.63	0.60
**RandF**	0.65	0.22	0.63	0.60	0.63

Note: Algorithms are sorted in descending order of ROC-AUC_test_.

Abbreviations: **BGLM**, Boosted Generalized Linear Model; **BCT**, Boosted Classification Tree; **SVM**, Support Vector Machines with Linear Kernel; **PLS**, Partial Least Squares; **GLMNET**, Lasso and Elastic-Net Regularized Generalized Linear Model; **PMR**, Penalized Multinomial Regression; **LogiR**, Logistic Regression; **xgboost**, Extreme Gradient Boosting; **CART**, Classification and Regression Trees; **RandF**, Random Forest; **ROC**, receiver operating characteristic; **AUC**, area under the curve; **ROC-AUC_test_**, ROC-AUC value on the test set.

### 3.4 Variable importance and feature coefficient of the best model

In our study, the BGLM model (ROC-AUC_test_ = 0.68) exhibited the best performance among the 10 models. Fig. A.2 presents the contributions of the 10 features, which are important for predicting exercise habit acquisition using the BGLM model. A change in lifestyle behavior based on the TTM (stage of action and maintenance) was the most important feature, followed by regular physical activity, normal HDL-C, and daily alcohol consumption of ≥60 g, all of which had an importance of >50 on a 100-point scale.

**Fig. 2:**
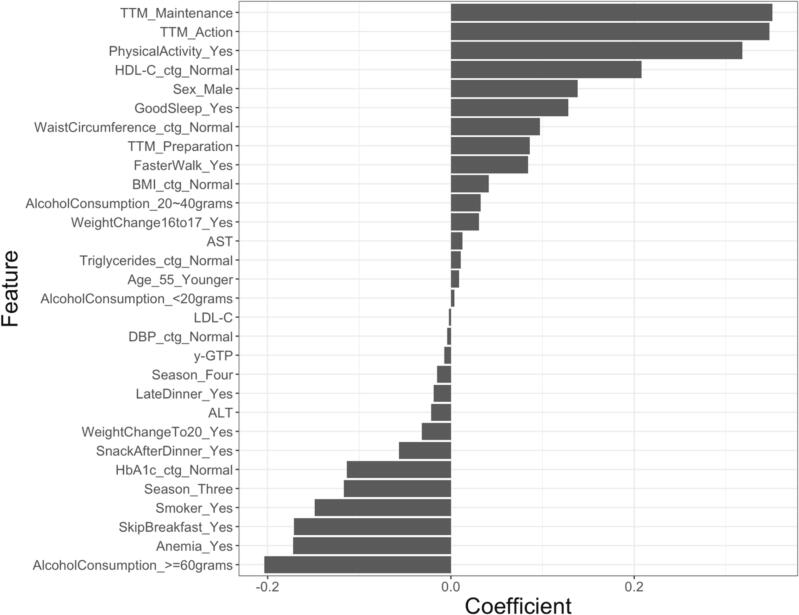
Feature coefficient in the Boosted Generalized Linear Model (BGLM) for Japanese workers at high risk for metabolic syndrome who received the Specific Health Guidance, 2017–2018. Abbreviations: **TTM**, transtheoretical model; **HDL-C**, high-density lipoprotein cholesterol; **BMI**, body mass index; **AST**, aspartate transaminase; **LDL-C**, low-density lipoprotein cholesterol; **LDL-C**, low-density lipoprotein cholesterol; **DBP**, diastolic blood pressure; **y-GTP**, gamma-glutamyl transpeptidase as well as shown as γ-GTP in the main text**; ALT**, alanine transaminase; **HbA1c**, hemoglobin A1c. Definitions: **TTM_Maintenance**, maintenance stage of TTM; **TTM_Action**, action stage of TTM; **PhysicalActivity_Yes**, whether walking or performing any equivalent amount of physical activity for more than one hour a day in daily life; **HDL-C_ctg_Normal**, normal HDL-C levels (< 40 mg/dL) based on the Specific Health Guidance (SHG) criteria; **TTM_Preparation**, preparation stage of TTM; **FasterWalk_Yes**, the participant thought his/her walking speed was faster than the speed of those of the same age and sex; **BMI_ctg_Normal,** normal BMI (< 25 kg/m^2^) based on the SHG criteria; **AlcoholConsumption_20 ∼ 40 g**, consuming alcohol 20–40 g per day; **WeightChange16to17_Yes,** body weight changes of more than three kilograms from 2016 to 2017; **Triglycerides_ctg_Normal,** normal triglycerides levels (≥ 150 mg/day) based on SHG criteria; **Age_55_Younger**, younger than 55 years of age; **AlcoholConsumption_<20 g,** consuming alcohol < 20 g per day; **DBP_ctg_Normal**, normal DBP (≥ 85 mmHg) based on the SHG criteria; **Season_Four**, the date of receiving SHG was during 12/1/2017–3/31/2018; **LateDinner_Yes**, eating supper two hours before bedtime more than three times a week; **WeightChangeTo20_Yes**, body weight gain of more than 10 kg from the age of 20; **SnackAfterDinner_Yes**, eating supper two hours before bedtime more than three times a week; **HbA1c_ctg_Normal**, normal HbA1c levels (< 5.6 %) based on SHG criteria; **Season_Three**, the date of receiving SHG was during 9/1/2017–11/30/2017; **Smoker_Yes**, had smoked a total of over 100 cigarettes or have smoked over a period of six months and have been smoking over the past month; **SkipBreakfast_Yes**, skipping breakfast more than three times a week; **Anemia_Yes**, had anemia; **AlcoholConsumption_>=60 g**, consuming alcohol ≥60 g per day.

Fig. 2 presents the coefficients of features using the BGLM model. In the BGLM model, the coefficients of the top five features were as follows: 0.35 for the action stage of change in lifestyle behavior based on TTM; 0.35 for the maintenance stage; 0.32 for physical activity; 0.21 for normal HDL-C; and −0.20 for daily alcohol consumption of ≥60 g.

## 4 Discussion

In the current study, we applied 10 algorithms to build prediction models for acquiring exercise habits among middle-aged Japanese workers. The BGLM exhibited the best performance in predicting exercise habit acquisition. Accordingly, the prediction model used in our study could be employed to identify individuals who are more likely to acquire exercise habits through MHG.

The prediction results revealed that the BGLM model outperformed the other models in predicting the likelihood that an individual would acquire exercise habits through the MHG, with the greatest ROC-AUC_test_ value of 0.68. However, the ROC-AUC_test_ value was judged as low. In a study on predicting type two diabetes, the highest ROC-AUC_test_ of the model constructed using xgboost was 0.78 (Liu et al., 2022). In another study for predicting diabetic nephropathy, the highest ROC-AUC_test_ of the model constructed using xgboost was 0.97 (Yin et al., 2024). In a study predicting future dementia and dementia subtypes, the ROC-AUC_test_ for predicting all-cause dementia, Alzheimer’s disease, and vascular dementia were 0.86, 0.86, and 0.87, respectively (Qiang et al., 2024). The low ROC-AUC_test_ indicates that variables in the current study were insufficient to adequately predict the acquisition of exercise habits. Various factors, such as income, education, interpersonal relationships, tiredness, and weakness, can also affect exercise or physical activity (Lee and Park, 2021, Trost et al., 2002). Although the participants in these studies were non-Japanese, the related factors are still worth considering in the future SHC.

Considering only 30 % of individuals in Japan had regular exercise habits (MHLW, 2020), it is essential to note that 9 % of the participants in the current study acquired exercise habits in the following year (Table A.3). Although the ROC-AUC_test_ was low, the top five important factors were associated with acquiring exercise habits and may serve as a critical reference for future personalized guidance.

The results of the variable importance analysis indicated that the stage of change in lifestyle behavior based on the TTM (stage of action and maintenance) was the most important feature, followed by physical activity, normal HDL-C levels, and daily alcohol consumption of ≥60 g. TTM is the conviction that one can accomplish specific goals (Bandura and Adams, 1977). TTM components include decisional balance, self-efficacy, change processes, and stages of change. A cross-sectional study has reported that the progression of the action and maintenance stage of TTM was consistent with health behavior, such as exercise, physical activity, and walking speed among participants of SHC (Mizoshita et al., 2011). Furthermore, individuals with higher self-efficacy may overcome barriers to exercise (Ayotte et al., 2010). As we excluded individuals with exercise habits at the baseline, the lifestyle behavior TTM was not related to exercise habits but rather to dietary or other habits. Nevertheless, lifestyle behavior TTM indicates the level of health awareness, and the analysis revealed that individuals who had already taken action or maintained other healthy lifestyle habits were more likely to acquire exercise habits. Physical activity was shown to be negatively associated with unhealthy eating behaviors (Martinez-Avila et al., 2020). We speculate that individuals who have other healthy lifestyle habits may have lower barriers to forming new healthy habits.

*The Physical Activity and Exercise Guide for Health Promotion* (MHLW, 2024) in Japan recommends that adults perform at least 60 min of physical activities above three metabolic equivalent of tasks (METs) per day (over 23 METs·hour per week), with particular emphasis on walking (over 8,000 steps per day) because it is an easy and straightforward measure. The findings of our study indicate that individuals who performed regular physical activities, including adequate walking, were more prone to acquiring exercise habits (coefficient = 0.32). Although exercise habits and physical activity or walking are associated with health benefits, the correlation between them is yet to be explored. Individuals with high physical activity levels may have fewer barriers to exercise and are more likely to begin exercising spontaneously. High physical activity also reflects healthier bodily function, allowing the body to adapt to exercise easily. Additional investigations are required to validate this hypothesis.

Normal HDL-C levels (coefficient = 0.17) were positively associated with exercise habits. A review that summarized the findings of nine prospective cohort studies found that physical activity (i.e., daily walking or aerobic exercise) could enhance HDL-C levels among the Japanese population (Koba et al., 2011). Zhuang et al. (2020) reported that HDL-C levels were higher in physically active individuals than in inactive individuals. Furthermore, HDL-C was shown to be associated with lifestyle habits, such as good sleep (Zhuang et al., 2020, Zhang et al., 2022) and a healthy diet (Lu et al., 2024). Normal HDL-C levels indicate that individuals had a healthier lifestyle, resulting in healthier bodily functions and making them less resistant to exercise, with suitable physiological conditions to start exercising. HDL-C may be used as a secondary reference to infer whether an individual is prone to acquiring exercise habits based on other important characteristics.

Interestingly, daily alcohol consumption of ≥60 g (coefficient = −0.20) was associated with the acquisition of exercise habits in our study. Several studies have confirmed the health risks associated with alcohol consumption (Hagstrom et al., 2024, Jun et al., 2023, Koyanagi et al., 2024, Kunutsor et al., 2024), which are well-recognized in the realm of health promotion. Liangpunsakul et al. (2010) revealed that physical activity was negatively associated with alcohol consumption in both males and females. Despite the significant association (P < 0.05), the results warrant further confirmation owing to the large deviation of alcohol consumption and small sample size of individuals with high consumption. The findings of the present study complement those of previous reports. Excessive alcohol consumption reflects a lack of health awareness, harms health, reduces bodily function, and makes it difficult to acquire exercise habits. Thus, alcohol consumption may lead to physiological conditions that hinder exercise performance. Future research is crucial to better understand the association between alcohol consumption and exercise habits.

Additionally, skipping breakfast and smoking are unhealthy lifestyle habits that negatively impact the acquisition of exercise habits. Anemia and sleep quality, as physiological conditions, also affected the acquisition of exercise habits. Notably, males were more likely to acquire exercise habits than females. These results can also serve as references for future MHG.

The strength of our study is the use of the machine learning approach. Although machine learning has been widely applied in healthcare, it is primarily applied for medical diagnosis, disease progression, and treatment prognosis prediction and less for health habits-related research. In the current study, an exploratory attempt was made to analyze factors affecting exercise habits. Although the constructed model was not ideal, we aimed to identify the relevant effective factors. Data from the SHC, collected for several years as a national-level health improvement policy in Japan, have high clinical value.

Nevertheless, this study has certain limitations. First, the ROC-AUC_test_ in this study was lower than that in previous studies, which suggests that the features utilized in this study may be inadequate to sufficiently interpret the reasons for the acquisition of exercise habits. Second, there were notable differences in several features between the individuals included and excluded from the analysis (P < 0.05), limiting the generalizability of our findings.

## 5 Conclusions

In the current study, we found that motivation to improve health habits, physical activity, skipping breakfast, and normal HDL-C levels were essential factors that influenced the acquisition of exercise habits. The lower ROC-AUC_test_ suggests that additional candidate variables are needed in future studies to predict the acquisition of exercise habits. The results of this study may provide reliable clinical recommendations for improving SHG levels.

## Funding sources

This work was supported by the 10.13039/100009619Japan Agency for Medical Research and Development (grant numbers 21ek0210124h9903 and JP23rea522107).

## CRediT authorship contribution statement

**Jiawei Wan:** Writing – original draft, Visualization, Software, Methodology, Formal analysis, Data curation, Conceptualization. **Kyohsuke Wakaba:** Writing – original draft, Visualization, Software, Methodology, Formal analysis, Data curation, Conceptualization. **Takeshi Onoue:** Writing – review & editing. **Kazuyo Tsushita:** Writing – review & editing, Resources, Project administration, Funding acquisition. **Yoshio Nakata:** Writing – review & editing, Validation, Supervision, Resources, Project administration, Methodology, Funding acquisition, Conceptualization.

## Declaration of competing interest

The authors declare the following financial interests/personal relationships which may be considered as potential competing interests: Yoshio Nakata reports financial support was provided by Japan Agency for Medical Research and Development. Kazuyo Tsushita reports financial support was provided by Japan Agency for Medical Research and Development. If there are other authors, they declare that they have no known competing financial interests or personal relationships that could have appeared to influence the work reported in this paper.

## Data Availability

Data will be made available on request.
