# Supplementary material for: Factors associated with acquiring exercise habits through health guidance for metabolic syndrome among middle-aged Japanese workers: A machine learning approach
Source: Prev Med Rep. 2024 Oct 19;48:102915. doi: 10.1016/j.pmedr.2024.102915 (PMC11544079; doi:10.1016/j.pmedr.2024.102915)
Supplement: Supplementary Data 1 [file mmc1.pdf]

**Table A.1** Specific Health Checkup Questionnaire items for Japanese workers at high risk for metabolic syndrome who received the Specific Health Guidance, 2017–2018.

| Features | Name in Coding | Questionnaire Content | Answer Options |
|---|---|---|---|
| **Anemia** | Anemia | Have you ever been diagnosed as anemic? | No, Yes |
| **Smoker** | Smoker | “A heavy smoker” refers to those who have smoked a total of over 100 cigarettes or have smoked over a period of six months and have been smoking over the past month. | No, Yes |
| **Body weight gain of at least 10 kilograms from the age of 20 years** | WeightChange-To20 | Have you gained over 10 kilograms from your weight at age 20? | No, Yes |
| **Physical activity** | PhysicalActivity | In your daily life, do you walk or do any equivalent amount of physical activity for more than one hour a day? | No, Yes |

| Features | Name in Coding | Questionnaire Content | Answer Options |
| --- | --- | --- | --- |
| **Walking speed** | FasterWalk | Is your walking speed faster than the speed of those of your age and sex? | No, Yes |
| **Body weight changes of more than three kilograms in the past year** | WeightChange-16to17 | Have there been any body weight changes in the past year? | No, Yes |
| **Eating speed** | EatSpeed | Is your eating speed quicker than others? | Fast, Normal or Slow* |
| **Late dinner** | LateDinner | Do you eat supper two hours before bedtime more than three times a week? | No, Yes |
| **Eating snacks after dinner** | SnackAfterDin-ner | Do you eat snacks after supper more than three times a week? | No, Yes |
| **Skipping breakfast** | SkipBreakfast | Do you skip breakfast more than three times a week? | No, Yes |

| Features | Name in Coding | Questionnaire Content | Answer Options |
|---|---|---|---|
| **Daily alcohol consumption** | AlcoholConsu-mption | How much do you drink per day?<br>One Gou***=Sake (180 ml), middle-size beer (500 ml), Shochu (80 ml), whisky (60 ml), or two glasses of wine (240 ml) | Don't drink**,<br>Less than one Gou,<br>One to two Gou,<br>Two to three Gou,<br>More than three Gou |
| **Good sleep quality** | GoodSleep | Do you sleep well and sufficiently? | No, Yes |
| **Lifestyle behavior based on the TTM** | TTM | Do you want to improve your eating and exercise habits? | Don't want: pre-contemplation****,<br>Do want: contemplation,<br>want to improve in near future(within a month) and began to start :preparation,<br>Already trying to improve (less than six months): action,<br>Already trying to improve (over six months): maintenance |

| Features | Name in Coding | Questionnaire Content | Answer Options |
|---|---|---|---|
| **Willingness to receive SHG** | GuidedWilling-ness | Do you want to use the opportunity of health instructions to improve your life habits? | No, Yes |
| **Exercise habits in 2018 (Outcome)** | ExerciseHabit_2018 | Are you in the habit of exercising to sweat lightly for over 30 minutes a time, two times weekly, for over a year? | No, Yes |

Abbreviation: **TTM**, transtheoretical model; **SHG**, Specific Health Checkups.

* As fewer people answered "Slow", "Slow" and "Normal" were integrated into one classification.

** "Don't drink" is the result of answering "Don't drink" in another question investigating the frequency of drinking.

*** Go, a measurement of alcohol consumption, 1 Go = 20 grams.

**** Words after ":" indicates the stages in the Transtheoretical Model.

**Table A.2** The R packages and instructions in brief of each algorithm used in this study.

| **Machine Learning Algorithms** | **Function {R package}** | **Version** | **Instruction** |
|---|---|---|---|
| Logistic Regression (LogiR) | glm {stats} | 4.3.1 | LogiR is a classical and commonly used machine learning algorithm that has been utilized in many studies. LogiR is similar to multivariate linear regression, given that its outcome is the result of the logarithmic transformation of its probabilities, and the regression coefficients are natural logarithms (Anderson et al., 2003). LogiR is based on the sigmoid function to measure the relationship between categorical dependent and independent variables (Choi et al., 2020). |
| Classification and Regression Trees (CART) | rpart {part} | 4.1.19 | CART is an algorithm capable of efficiently processing binary classification problems. CART can quickly partition a dataset into two distinct nodes and repeat the splitting of the nodes continuously until the purest node is split or a specified requirement is met (e.g., minimum number of splitting and maximum depth) (Breiman et al., 1984). |

| Machine Learning Algorithms | Function {R package} | Version | Instruction |
|---|---|---|---|
| Lasso and Elastic-Net Regularized Generalized Linear Model (GLMNET) | glmnet {glmnet} | 4.1-8 | GLMNET is an algorithm for fitting generalized linear and similar models using penalized maximum likelihood estimation. GLMNET combines both least absolute shrinkage and selection operator (LASSO) regression and ridge regression, and by adjusting the elastic net mixing parameter, the model could have various fitting patterns (Friedman et al., 2010). |
| Penalized Multinomial Regression (PMR) | multinom {nnet} | 7.3-19 | PMR is an algorithm that processes multilevel categorical variables but can still process binomial categorization. It is computed as in logistic regression. Given that PMR is a penalized linear model with ridge regression regularization to prevent overfitting, it was considered to result in better predictive performance than logistic regression (Venables and Ripley, 2002). |
| Extreme Gradient Boosting (xgboost) | xgbTree {xgboost} | 1.7.5.1 | Xgboost is a very popular machine learning algorithm based on gradient-boosted trees, where the optimal solution is derived through multiple iterations. Xgboost is very efficient and builds highly robust and highly accurate stable models through a series of weak learners. Xgboost combines LASSO and ridge regression regularizations into the objective function, which allows the model to avoid overfitting as much as possible and obtain accurate and generic predictive results (Chen et al., 2023). |

| **Machine Learning Algorithms** | **Function {R package}** | **Version** | **Instruction** |
| --- | --- | --- | --- |
| Random Forest (RandF) | ranger {ranger} | 0.15.1 | RandF is an integrated algorithm that comprises a number of decision trees. The main concept is to generate decision trees for different subsets of the same dataset by randomly sampling different variables in one dataset. RandF generates the final prediction model by selecting the majority of the decision tree classification results. The sampling within the algorithm allows RandF to avoid overfitting to obtain generalized predictive ability (Wright et al., 2017). |
| Boosted Generalized Linear Model (BGLM) | glmboost {mboost} | 2.9-8 | The BGLM is markedly similar to the generalized linear model, with the only difference being the addition of a Gradient-Boosting algorithm to perform feature selection (Hofner et al., 2014). |
| Support Vector Machines with Linear Kernel (SVM) | svmLinear {kernlab} | 0.9-32 | SVM is widely used in the biomedical field. SVM is good at binary classification problems and is able to find decision boundaries for different classes of data. When using the kernel trick, SVM with kernel tricks can project the data into a high-dimensional feature space for data classification. The use of the kernel trick allows the classifier to reduce computational cost by eliminating the need to compute the coordinates of the data distribution in space (Karatzoglou et al., 2004). |

| Machine Learning Algorithms | Function {R package} | Version | Instruction |
|---|---|---|---|
| Partial Least Squares (PLS) | kernelpls {pls} | 2.8-3 | PLS is an algorithm that builds a least squares regression model by projecting data into a new feature space. In this study, the projection method used is the kernel trick. PLS is characterized by its ability to effectively deal with the problem of multicollinearity between independent variables (Liland et al., 2023). |
| Boosted Classification Tree (BCT) | ada {ada} | 2.0-5 | BCT is an integrated tree model based on the Gradient Boosting algorithm. Gradient Boosting is an algorithm that iteratively trains multiple weak learners and distributes higher weights to weak learners with high classification error rates to enhance training in the next iteration, finally integrating each weak learner according to its weight. In BCT, the base learners are CART models (Culp et al., 2016). |

**Table A.3** Characteristics of Japanese workers at high risk for metabolic syndrome who received the Specific Health Guidance, by inclusion status, 2017–2018.

| | All<br>n = 24,930 | Excluded<br>n = 8,459 | Included<br>n = 16,471 | P value |
|---|---|---|---|---|
| **Sex, n (%):** | | | | <0.01 |
| **Female** | 6,632 (26.6) | 2,163 (25.6) | 4,469 (27.1) | |
| **Male** | 18,298 (73.4) | 6,296 (74.4) | 12,002 (72.9) | |
| **Season, n (%):** | | | | <0.01 |
| **One** | 6,328 (25.4) | 1,619 (19.1) | 4,709 (28.6) | |
| **Two** | 7,043 (28.3) | 2,358 (27.9) | 4,685 (28.4) | |
| **Three** | 6,107 (24.5) | 2,226 (26.3) | 3,881 (23.6) | |
| **Four** | 5,452 (21.9) | 2,256 (26.7) | 3,196 (19.4) | |
| **Age, year** | 49.9 (6.4) | 50.6 (6.8) | 49.5 (6.2) | <0.01 |
| **Height, cm** | 167.5 (8.6) | 167.4 (8.6) | 167.6 (8.6) | 0.30 |
| **Body weight, kg** | 74.2 (9.3) | 73.9 (9.2) | 74.4 (9.3) | <0.01 |
| **Waist circumference, cm** | 90.2 (6.7) | 89.8 (6.6) | 90.5 (6.7) | <0.01 |
| **BMI, $kg/m^2$** | 26.4 (2.7) | 26.4 (2.7) | 26.5 (2.7) | <0.01 |

| | All<br>n = 24,930 | Excluded<br>n = 8,459 | Included<br>n = 16,471 | P value |
|---|---|---|---|---|
| **SBP, mmHg** | 127.5 (14.7) | 128.2 (14.6) | 127.1 (14.8) | <0.01 |
| **DBP, mmHg** | 80.1 (10.7) | 80.4 (10.5) | 80.0 (10.8) | <0.01 |
| **AST, U/L** | 23.9 (10.7) | 23.7 (10.6) | 23.9 (10.8) | 0.14 |
| **ALT, U/L** | 29.1 (21.8) | 28.9 (24.9) | 29.2 (20.0) | 0.25 |
| **γ-GTP, U/L** | 46.3 (46.0) | 45.0 (44.6) | 47.0 (46.7) | <0.01 |
| **Triglycerides, ㎎/dL** | 126.8 (84.9) | 124.6 (87.4) | 127.9 (83.6) | <0.01 |
| **HDL-C, ㎎/dL** | 57.8 (13.9) | 58.7 (14.1) | 57.4 (13.8) | <0.01 |
| **LDL-C, ㎎/dL** | 133.1 (29.8) | 133.2 (29.6) | 133.1 (29.8) | 0.86 |
| **HbA1c, %** | 5.6 (0.5) | 5.6 (0.5) | 5.6 (0.4) | <0.01 |
| **Anemia, n (%):** | | | | <0.01 |
| **No** | 20,905 (92.8) | 5,526 (91.2) | 15,379 (93.4) | |
| **Yes** | 1,625 (7.2) | 533 (8.8) | 1,092 (6.6) | |
| **Smoker, n (%):** | | | | <0.05 |
| **No** | 23,959 (96.1) | 8,100 (95.8) | 15,859 (96.3%) | |
| **Yes** | 971 (3.9) | 359 (4.2) | 612 (3.7%) | |

| | All<br>n = 24,930 | Excluded<br>n = 8,459 | Included<br>n = 16,471 | P value |
|---|---|---|---|---|
| **Body weight gain of ≥ 10 kilograms from the age of 20 years, n (%):** | | | | <0.01 |
| **No** | 7,444 (30.1) | 2,814 (34.2) | 4,630 (28.1) | |
| **Yes** | 17,251 (69.9) | 5,410 (65.8) | 11,841 (71.9) | |
| **Regular physical activity or walking at least one hour per day, n (%):** | | | | <0.01 |
| **No** | 17,760 (71.9) | 5,574 (67.8) | 12,186 (74.0) | |
| **Yes** | 6,927 (28.1) | 2,642 (32.2) | 4,285 (26.0) | |
| **Faster walking speed than others, n (%):** | | | | 0.08 |
| **No** | 13,454 (60.7) | 3,403 (59.7) | 10,051 (61.0) | |
| **Yes** | 8,717 (39.3) | 2,297 (40.3) | 6,420 (39.0) | |
| **Body weight changes of more than three** | | | | <0.01 |

| | All<br>n = 24,930 | Excluded<br>n = 8,459 | Included<br>n = 16,471 | P value |
|---|---|---|---|---|
| **kilograms from 2016 to 2017, n (%):** | | | | |
| **No** | 15,289 (65.8) | 4,767 (70.3) | 10,522 (63.9) | |
| **Yes** | 7,961 (34.2) | 2,012 (29.7) | 5,949 (36.1) | |
| **Eating speed, n (%):** | | | | <0.01 |
| **Fast** | 9,881 (40.0) | 3,432 (41.7) | 6,449 (39.2) | |
| **Normal or slow** | 14,824 (60.0) | 4,802 (58.3) | 10,022 (60.8) | |
| **Late dinner time, n (%):** | | | | <0.01 |
| **No** | 15,888 (64.2) | 5,514 (66.7) | 10,374 (63.0) | |
| **Yes** | 8,844 (35.8) | 2,747 (33.3) | 6,097 (37.0) | |
| **Eating snacks after dinner, n (%):** | | | | 0.11 |
| **No** | 20,256 (82.1) | 6,683 (81.6) | 13,573 (82.4) | |
| **Yes** | 4,409 (17.9) | 1,511 (18.4) | 2,898 (17.6) | |
| **Skipping breakfast, n (%):** | | | | <0.01 |

| | All<br>n = 24,930 | Excluded<br>n = 8,459 | Included<br>n = 16,471 | P value |
|---|---|---|---|---|
| **No** | 20,794 (84.1) | 7,035 (85.2) | 13,759 (83.5) | |
| **Yes** | 3,930 (15.9) | 1,218 (14.8) | 2,712 (16.5) | |
| **Daily alcohol consumption, n (%):** | | | | <0.01 |
| **Don’t drink** | 5,819 (23.3) | 2,299 (27.2) | 3,520 (21.4) | |
| **Less than 20 grams** | 8,446 (33.9) | 2,536 (30.0) | 5,910 (35.9) | |
| **20–40 grams** | 6,329 (25.4) | 2,002 (23.7) | 4,327 (26.3) | |
| **40–60 grams** | 3,117 (12.5) | 1,147 (13.6) | 1,970 (12.0) | |
| **More than 60 grams** | 1,219 (4.9) | 475 (5.6) | 744 (4.5) | |
| **Good sleep quality, n (%):** | | | | <0.01 |
| **No** | 10,424 (42.3) | 2,910 (35.5) | 7,514 (45.6) | |
| **Yes** | 14,246 (57.7) | 5,289 (64.5) | 8,957 (54.4) | |
| **Lifestyle behavior based on the TTM, n (%):** | | | | <0.05 |
| **Precontemplation** | 4,238 (19.1) | 1,121 (19.7) | 3,117 (18.9) | |
| **Contemplation** | 9,807 (44.3) | 2,452 (43.1) | 7,355 (44.7) | |

| | All<br>n = 24,930 | Excluded<br>n = 8,459 | Included<br>n = 16,471 | P value |
|---|---|---|---|---|
| **Preparation** | 3,607 (16.3) | 897 (15.8) | 2,710 (16.5) | |
| **Action** | 2,461 (11.1) | 655 (11.5) | 1,806 (11.0) | |
| **Maintenance** | 2,046 (9.2) | 563 (9.9) | 1,483 (9.0) | |
| **Willingness to receive SHG, n (%):** | | | | 0.17 |
| **No** | 16,149 (72.8) | 4,118 (72.1%) | 12,031 (73.0) | |
| **Yes** | 6,034 (27.2) | 1,594 (27.9%) | 4,440 (27.0) | |
| **Exercise habits in 2018, n (%):** | | | | 0.36 |
| **No** | 19,350 (91.0) | 4,386 (91.3) | 14,964 (90.9) | |
| **Yes** | 1,925 (9.0) | 418 (8.7) | 1,507 (9.1) | |

Note: Data are shown as the mean (standard deviation) for continuous variables or n (%) for categorical variables. P values were generated using Welch's t-test or the chi-squared test. Table A.1 in the appendix contains detailed definitions of the questionnaire variables.

Abbreviations: **BMI**, body mass index; **SBP**, systolic blood pressure; **DBP**, diastolic blood pressure; **AST**, aspartate transaminase; **ALT**, alanine transaminase; **γ-GTP,** gamma-glutamyl transpeptidase; **HDL-C**, high-density lipoprotein cholesterol; **LDL-C**, low-density lipoprotein cholesterol; **HbA1c**, hemoglobin A1c; **TTM**, transtheoretical model; **SHG**, Specific Health Guidance.

Definitions: **Season**, the dates of receiving SHG were categorized into four seasons (Season One: 4/1/2017–5/31/2017, Season Two: 6/1/2017–8/31/2017, Season Three: 9/1/2017–11/30/2017, Season Four: 12/1/2017–3/31/2018); **Smoker**, had smoked a total of over 100 cigarettes or have smoked over a period of six months and have been smoking over the past month; **Regular physical activity or walking at least one hour per day**, whether walking or performing any equivalent amount of physical activity for more than one hour a day in daily life; **Faster walking speed than others**, whether the participant thought his/her walking speed was faster than the speed of those of the same age and sex; **Eating speed**, whether the participant thought he/she ate faster than others; **Late dinner time**, eating supper two hours before bedtime more than three times a week; **Eating snacks after dinner**, eating snacks after supper more than three times a week; **Skipping breakfast**, skipping breakfast more than three times a week; **Good sleep quality**, whether the participant felt he/she slept well and sufficiently; **Willingness to receive SHG**, whether the participant wanted to use the opportunity of health instructions for improvement of his/her life habits; **Exercise habits**, participation in any physical activity for at least 30 minutes/time and two days/week that caused light sweating in the past 12 months.

**Table A.4** LASSO regression and variable names in coding for Japanese workers at high risk for metabolic syndrome who received the Specific Health Guidance, 2017–2018.

| Candidate variables | Name in coding | Coefficient |
|---|---|---|
| Exercise habits in 2018 | ExerciseHabit_2018 (outcome) | - |
| Height | Height | 0.00 |
| Body weight | Weight | 0.03 |
| AST | AST | 0.16 |
| ALT | ALT | -0.16 |
| γ-GTP | y-GTP | +0.00 |
| LDL-C | LDL-C | -0.01 |
| Sex: male | Sex_Male | 0.19 |
| Season to receive the guidance: Season Two | Season_Two | 0.03 |
| Season to receive the guidance: Season Three | Season_Three | -0.12 |
| Season to receive the guidance: Season Four | Season_Four | -0.00 |
| Anemia: Yes | Anemia_Yes | -0.17 |

| Candidate variables | Name in coding | Coefficient |
|---|---|---|
| Smoker: Yes | Smoker_Yes | -0.10 |
| Body weight gain of ≥ 10 kilograms from the age of 20 years: Yes | WeightChangeTo20_Yes | -0.15 |
| Regular physical activity or walking at least one hour per day in daily life: Yes | PhysicalActivity_Yes | 0.63 |
| Faster walking speed than others: yes | FasterWalk_Yes | 0.18 |
| Body weight changes of more than three kilograms from 2016 to 2017: Yes | WeightChange16to17_Yes | 0.13 |
| Eating speed: normal or slow | EatSpeed_NormalOrSlow | -0.03 |
| Late dinner time: Yes | LateDinner_Yes | -0.02 |
| Eating snacks after dinner: Yes | SnackAfterDinner_Yes | 0.02 |
| Skipping breakfast: Yes | SkipBreakfast_Yes | -0.17 |
| Daily alcohol consumption: Less than 20 grams | AlcoholConsumption_<20grams | 0.09 |
| Daily alcohol consumption: 20–40 grams | AlcoholConsumption_20~40grams | 0.12 |
| Daily alcohol consumption: 40–60 grams | AlcoholConsumption_40~60grams | 0.08 |

| Candidate variables | Name in coding | Coefficient |
|---|---|---|
| Daily alcohol consumption: More than 60 grams | AlcoholConsumption_>=60grams | -0.18 |
| Good sleep quality: Yes | GoodSleep_Yes | 0.16 |
| Lifestyle behavior based on the TTM: Contemplation | TTM_Contemplation | 0.14 |
| Lifestyle behavior based on the TTM: Preparation | TTM_Preparation | 0.41 |
| Lifestyle behavior based on the TTM: Action | TTM_Action | 0.91 |
| Lifestyle behavior based on the TTM: Maintenance | TTM_Maintenance | 0.90 |
| Willingness to receive SHG: Yes | GuidedWillingness_Yes | 0.03 |
| Age: younger than 55 years | Age_55_Younger | -0.07 |
| Waist circumference (SHG criteria): Normal | WaistCircumference_ctg_Normal* | 0.21 |
| BMI (SHG criteria): Normal | BMI_ctg_Normal* | 0.07 |
| SBP (SHG criteria): Normal | SBP_ctg_Normal* | 0.02 |
| DBP (SHG criteria): Normal | DBP_ctg_Normal* | -0.07 |
| Triglycerides (SHG criteria): Normal | Triglycerides_ctg_Normal* | 0.02 |

| Candidate variables | Name in coding | Coefficient |
|---|---|---|
| HDL-C (SHG criteria): Normal | HDL-C_ctg_Normal* | 0.19 |
| HbA1c (SHG criteria): Normal | HbA1c_ctg_Normal* | -0.19 |

Note: String "ctg" means variables categorized according to SHG criteria. The values +0.00 and -0.00 indicate that the results of variable selection were not exactly zero, thus left in the analysis; +0.00 indicates a result slightly greater than zero, while -0.00 indicates a result slightly less than zero.

Abbreviations: **AST**, aspartate transaminase; **ALT**, alanine transaminase; **γ-GTP**, gamma-glutamyl transpeptidase; **LDL-C**, low-density lipoprotein cholesterol; **TTM**, transtheoretical model; **SHG**, specific health guidance; **BMI**, body mass index; **SBP**, systolic blood pressure; **DBP**, diastolic blood pressure; **HDL-C**, high-density lipoprotein cholesterol; **HbA1c**, hemoglobin A1c.

Definitions: **Season**, the dates of receiving SHG were categorized into four seasons (Season One: 4/1/2017–5/31/2017, Season Two: 6/1/2017–8/31/2017, Season Three: 9/1/2017–11/30/2017, Season Four: 12/1/2017–3/31/2018); **Smoker**, had smoked a total of over 100 cigarettes or have smoked over a period of six months and have been smoking over the past month; **Regular physical activity or walking at least one hour per day**, whether walking or any equivalent amount of physical activity was performed for more than one hour a day in daily life; **Faster walking speed than others**, whether the participant thought his/her walking speed was faster than the speed of those of the same age and sex; **Eating speed**, whether the participant thought he/she ate faster than others; **Late dinner time**, eating supper two hours before bedtime more than three times a week; **Eating snacks after dinner**, eating snacks after supper more than three times a week; **Skipping breakfast**, skipping breakfast more than three times a week; **Good sleep quality**, whether the participant felt he/she slept well and sufficiently; **Willingness to receive SHG**,

whether the participant wanted to use the opportunity of health instructions for improvement of his/her life habits; **BMI (SHG criteria): Normal**, BMI < 25 kg/$m^2$; **SBP (SHG criteria): Normal**, SBP < 130 mmHg; **DBP (SHG criteria): Normal**, DBP < 85 mmHg; **Triglycerides (SHG criteria): Normal**, triglycerides < 150 mg/dL; **HDL-C (SHG criteria): Normal**, HDL-C < 40 mg/dL; **HbA1c (SHG criteria)**: **Normal**, HbA1c < 5.6%.

**Table A.5** Multicollinearity assessment for variables used in modeling results for Japanese workers at high risk for metabolic syndrome who received the Specific Health Guidance, 2017–2018.

| **Candidate variable** | **Name in Coding** | **VIF** |
|---|---|---|
| Body weight | Weight | 1.92 |
| AST | AST | 3.14 |
| ALT | ALT | 3.39 |
| γ-GTP | y-GTP | 1.34 |
| LDL-C | LDL-C | 2.00 |
| Sex: male | Sex_Male | 1.08 |
| Season to receive the guidance: Season Two | Season_Two | 1.85 |
| Season to receive the guidance: Season Three | Season_Three | 1.45 |
| Season to receive the guidance: Season Four | Season_Four | 1.41 |
| Anemia: Yes | Anemia_Yes | 1.42 |
| Smoker: Yes | Smoker_Yes | 1.12 |

| **Candidate variable** | **Name in Coding** | **VIF** |
|---|---|---|
| Body weight gain of ≥ 10 kilograms from the age of 20 years: Yes | WeightChangeTo20_Yes | 1.18 |
| Regular physical activity or walking at least one hour per day in daily life: Yes | PhysicalActivity_Yes | 1.11 |
| Faster walking speed than others: yes | FasterWalk_Yes | 1.04 |
| Body weight changes of more than three kilograms from 2016 to 2017: Yes | WeightChange16to17_Yes | 1.07 |
| Eating speed: normal or slow | EatSpeed_NormalOrSlow | 1.08 |
| Late dinner time: Yes | LateDinner_Yes | 1.08 |
| Eating snacks after dinner: Yes | SnackAfterDinner_Yes | 1.13 |
| Skipping breakfast: Yes | SkipBreakfast_Yes | 1.05 |
| Daily alcohol consumption: Less than 20 grams | AlcoholConsumption_<20grams | 1.06 |
| Daily alcohol consumption: 20–40 grams | AlcoholConsumption_20~40grams | 1.93 |
| Daily alcohol consumption: 40–60 grams | AlcoholConsumption_40~60grams | 2.00 |

| Candidate variable | Name in Coding | VIF |
| --- | --- | --- |
| Daily alcohol consumption: More than 60 grams | AlcoholConsumption_>=60grams | 1.67 |
| Good sleep quality: Yes | GoodSleep_Yes | 1.23 |
| Lifestyle behavior based on the TTM: Contemplation | TTM_Contemplation | 1.03 |
| Lifestyle behavior based on the TTM: Preparation | TTM_Preparation | 2.55 |
| Lifestyle behavior based on the TTM: Action | TTM_Action | 2.04 |
| Lifestyle behavior based on the TTM: Maintenance | TTM_Maintenance | 2.04 |
| Willingness to receive SHG: Yes | GuidedWillingness_Yes | 1.91 |
| Age: younger than 55 years | Age_55_Younger | 1.04 |
| Waist circumference (SHG criteria): Normal | WaistCircumference_ctg_Normal | 1.11 |
| BMI (SHG criteria): Normal | BMI_ctg_Normal | 1.78 |
| SBP (SHG criteria): Normal | SBP_ctg_Normal | 1.47 |
| DBP (SHG criteria): Normal | DBP_ctg_Normal | 1.68 |
| Triglycerides (SHG criteria): Normal | Triglycerides_ctg_Normal | 1.56 |

| Candidate variable | Name in Coding | VIF |
|---|---|---|
| HDL-C (SHG criteria): Normal | HDL-C_ctg_Normal | 2.13 |
| HbA1c (SHG criteria): Normal | HbA1c_ctg_Normal | 1.16 |

Note: String "ctg" means variables categorized according to SHG criteria.

Abbreviations: **VIF**, variance inflation factor; **AST**, aspartate transaminase; **ALT**, alanine transaminase; **γ-GTP**, gamma-glutamyl transpeptidase; **LDL-C**, low-density lipoprotein cholesterol; **TTM**, transtheoretical model; **SHG**, specific health guidance; **BMI**, body mass index; **SBP**, systolic blood pressure; **DBP**, diastolic blood pressure; **HDL-C**, high-density lipoprotein cholesterol; **HbA1c**, hemoglobin A1c.

Definitions: **Season**, the dates of receiving SHG were categorized into four seasons (Season One: 4/1/2017–5/31/2017, Season Two: 6/1/2017–8/31/2017, Season Three: 9/1/2017–11/30/2017, Season Four: 12/1/2017–3/31/2018); **Smoker**, had smoked a total of over 100 cigarettes or have smoked over a period of six months and have been smoking over the past month; **Regular physical activity or walking at least one hour per day**, whether walking or any equivalent amount of physical activity was performed for more than one hour a day in daily life; **Faster walking speed than others**, whether the participant thought his/her walking speed was faster than the speed of those of the same age and sex; **Eating speed**, whether the participant thought he/she ate faster than others; **Late dinner time**, eating supper two hours before bedtime more than three times a week; **Eating snacks after dinner**, eating snacks after supper more than three times a week; **Skipping breakfast**, skipping breakfast more than three times a week; **Good sleep quality**, whether the participant felt he/she slept well and sufficiently; **Willingness to receive SHG**, whether the participant wanted to use the opportunity of health instructions for improvement of his/her life habits; **BMI (SHG criteria): Normal**, BMI < 25 $kg/m^2$; **SBP (SHG criteria): Normal**, SBP < 130 mmHg; **DBP (SHG criteria): Normal**, DBP < 85 mmHg; **Triglycerides**

**(SHG criteria): Normal**, triglycerides < 150 mg/dL; **HDL-C (SHG criteria): Normal**, HDL-C < 40 mg/dL; **HbA1c (SHG criteria): Normal**, HbA1c < 5.6%.

**Table A.6** Selected hyperparameters for machine learning models for Japanese workers at high risk for metabolic syndrome who received the Specific Health Guidance, 2017–2018.

| **Algorithm** | **Function** | **Hyperparameter** | **ROC-AUC** | **Sensitivity** | **Specificity** |
|---|---|---|---|---|---|
| LogiR | glm | None | 0.64 (0.02) | 0.64 (0.01) | 0.58 (0.04) |
| CART | rpart | cp=0.000905 | 0.59 (0.03) | 0.95 (0.01) | 0.09 (0.03) |
| GLMNET | glmnet | alpha=0.325 | 0.65 (0.02) | 0.64 (0.01) | 0.58 (0.05) |
| | | lambda=0.000141325387709475 | | | |
| PMR | multinom | decay=0.1 | 0.65 (0.02) | 0.64 (0.02) | 0.58 (0.04) |
| RandF | ranger | mtry=2 | 0.62 (0.02) | 1.00 (0.00) | 0.00 (0.01) |
| | | min.node.size=1 | | | |
| | | splitrule=gini | | | |
| SVM | svmLinear | C=1 | 0.65 (0.02) | 0.65 (0.01) | 0.57 (0.05) |
| PLS | kernelpls | ncomp=4 | 0.64 (0.02) | 0.65 (0.02) | 0.57 (0.04) |
| BCT | ada | nu=0.1 | 0.63 (0.03) | 0.71 (0.02) | 0.47 (0.05) |
| | | maxdepth=1 | | | |
| | | iter=100 | | | |

| Algorithm | Function | Hyperparameter | ROC-AUC | Sensitivity | Specificity |
|---|---|---|---|---|---|
| xgboost | xgbTree | eta=0.4<br>max_depth=1<br>gamma=0<br>colsample_bytree=0.8<br>min_child_weight=1<br>subsample=1<br>nrounds=250 | 0.64 (0.03) | 1.00 (0.00) | 0.01 (0.01) |
| BGLM | glmboost | mstop=250<br>prune=no | 0.64 (0.03) | 0.66 (0.02) | 0.56 (0.05) |

Note: ROC-AUC, sensitivity, and specificity on the test set are presented as the mean value (standard deviation). The hyperparameters were the results after tuning by 10-fold cross validation.

Abbreviations: **ROC**, receiver operating characteristic; **AUC**, area under the curve; **LogiR**, logistic regression; **CART**, classification and regression trees; **GLMNET**, lasso and elastic-net regularized generalized linear model; **PMR**, penalized multinomial regression; **RandF**, random forest; **SVM**, support vector machines with linear kernel; **PLS**, partial least squares; **BCT**, boosted classification tree; **xgboost**, extreme gradient boosting; **BGLM**, boosted generalized linear model.

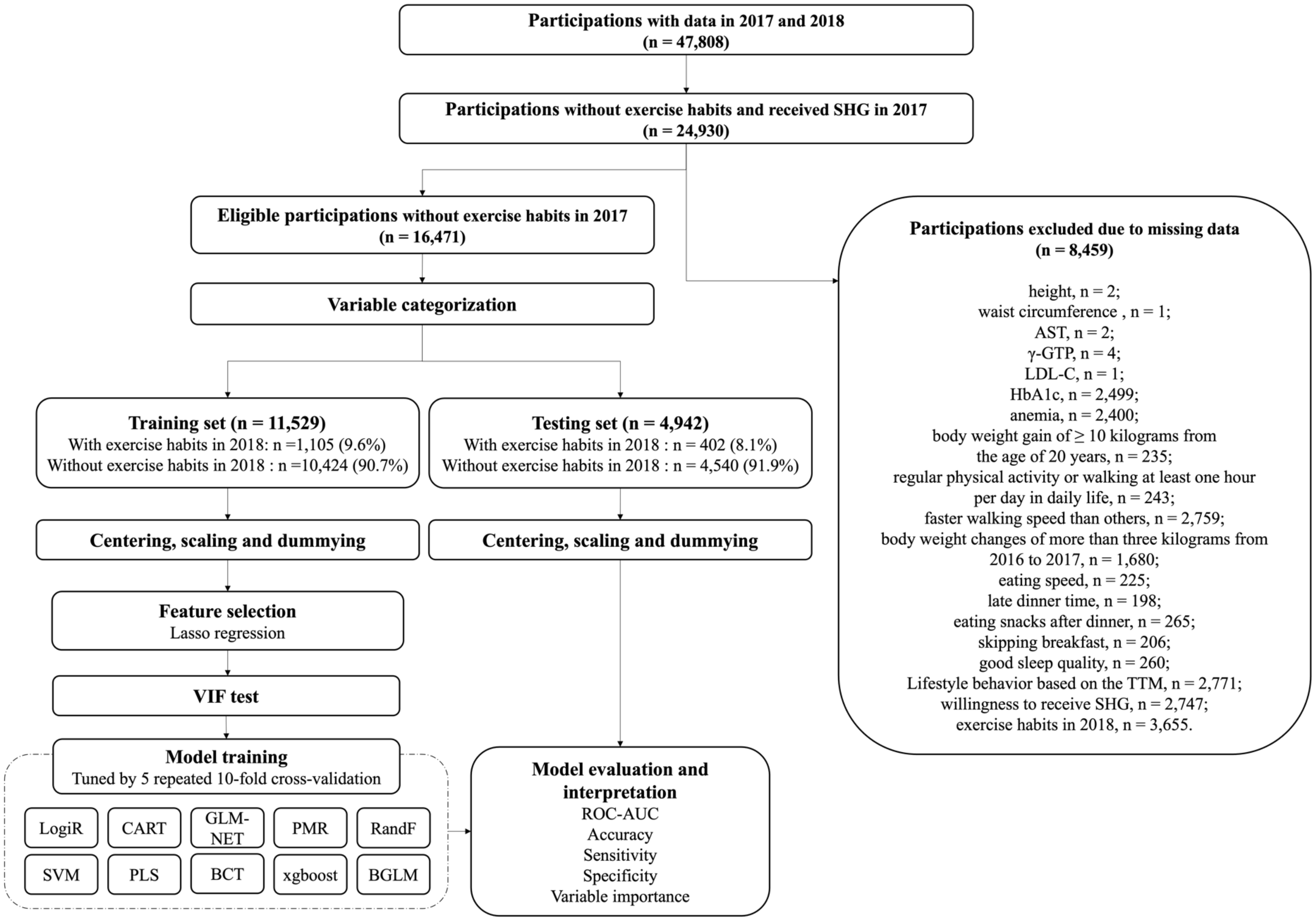
Participations with data in 2017 and 2018
(n = 47,808)
Participations without exercise habits and received SHG in 2017
(n = 24,930)
Eligible participations without exercise habits in 2017
(n = 16,471)
Participations excluded due to missing data
(n = 8,459)
height, n = 2;
waist circumference , n = 1;
AST, n = 2;
γ-GTP, n = 4;
LDL-C, n = 1;
HbA1c, n = 2,499;
anemia, n = 2,400;
body weight gain of ≥ 10 kilograms from the age of 20 years, n = 235;
regular physical activity or walking at least one hour per day in daily life, n = 243;
faster walking speed than others, n = 2,759;
body weight changes of more than three kilograms from 2016 to 2017, n = 1,680;
eating speed, n = 225;
late dinner time, n = 198;
eating snacks after dinner, n = 265;
skipping breakfast, n = 206;
good sleep quality, n = 260;
Lifestyle behavior based on the TTM, n = 2,771;
willingness to receive SHG, n = 2,747;
exercise habits in 2018, n = 3,655.
Variable categorization
Training set (n = 11,529)
With exercise habits in 2018: n =1,105 (9.6%)
Without exercise habits in 2018 : n =10,424 (90.7%)
Testing set (n = 4,942)
With exercise habits in 2018 : n = 402 (8.1%)
Without exercise habits in 2018 : n = 4,540 (91.9%)
Centering, scaling and dummying
Centering, scaling and dummying
Feature selection
Lasso regression
VIF test
Model training
Tuned by 5 repeated 10-fold cross-validation
LogiR
CART
GLM-NET
PMR
RandF
SVM
PLS
BCT
xgboost
BGLM
Model evaluation and interpretation
ROC-AUC
Accuracy
Sensitivity
Specificity
Variable importance

**Fig. A.1** Flowchart of analyzing factors associated with acquiring exercise habits among Japanese workers at high risk for metabolic syndrome who received the Specific Health Guidance, 2017–2018.

Abbreviations: **AST**, aspartate transaminase; **γ-GTP**, gamma-glutamyl transpeptidase; **LDL-C**, low-density lipoprotein cholesterol; **HbA1c**, hemoglobin A1c; **TTM**, transtheoretical model; **VIF test**, variance inflation factor test; **LogiR**, logistic regression; **CART**, classification and regression trees; **GLMNET**, lasso and elastic-net regularized generalized linear model; **PMR**, penalized multinomial regression; **RandF**, random forest; **SVM**, support vector machine with linear kernel; **PLS**, partial least squares; **BCT**, boosted classification tree; **xgboost**, Extreme Gradient Boosting; **BGLM**, boosted generalized Linear model; **ROC**, receiver operating characteristic; **AUC**, area under the curve.

Definitions: **Regular physical activity or walking at least one hour per day**, whether walking or performing any equivalent amount of physical activity for more than one hour a day in daily life; **Faster walking speed than others**, whether the participant thought his/her walking speed was faster than the speed of those of the same age and sex; **Eating speed**, whether the participant thought he/she ate faster than others; **Late dinner time**, eating supper two hours before bedtime more than three times a week; **Eating snacks after dinner**, eating snacks after supper more than three times a week; **Skipping breakfast**, skipping breakfast more than three times a week; **Good sleep quality**, whether the participant felt he/she slept well and sufficiently; **Willingness to receive SHG**, whether the participant wanted to use the opportunity of health instructions for improvement of his/her life habits; **Exercise habits**, participation in any physical activity for at least 30 minutes/time and two days/week that caused light sweating in the past 12 months.

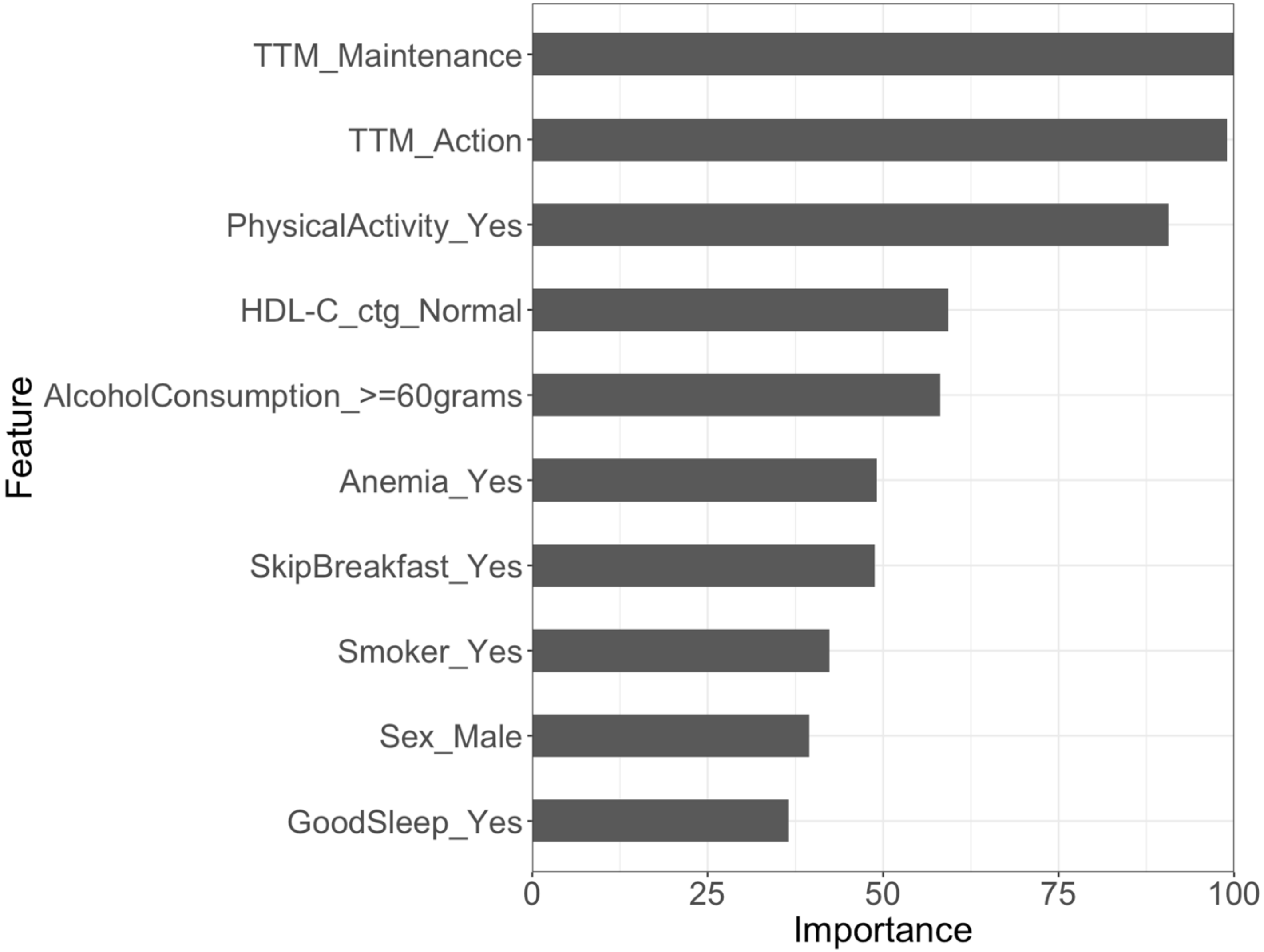
TTM_Maintenance
TTM_Action
PhysicalActivity_Yes
HDL-C_ctg_Normal
AlcoholConsumption_>=60grams
Anemia_Yes
SkipBreakfast_Yes
Smoker_Yes
Sex_Male
GoodSleep_Yes
Feature
0
25
50
75
100
Importance

**Fig. A.2** Feature importance in the Boosted Generalized Linear Model (BGLM) for Japanese workers at high risk for metabolic syndrome who received the Specific Health Guidance, 2017–2018.

Abbreviations: **TTM**, transtheoretical model; **HDL-C**, high-density lipoprotein cholesterol.

Definitions: **TTM_Maintenance**, maintenance stage of TTM; **TTM_Action**, action stage of TTM; **PhysicalActivity_Yes**, whether walking or any equivalent amount of physical activity was performed for more than one hour a day in daily life; **HDL-C_ctg_Normal**, normal HDL-C levels (< 40 mg/dL) based on the Specific Health Guidance (SHG) criteria; **AlcoholConsumption_>=60grams**, consuming alcohol ⩾ 60 grams per day; **Anemia_Yes**, had anemia; **SkipBreakfast_Yes**, skipping breakfast more than three times a week; **Smoke_Yes**, Smoke, had smoked a total of over 100 cigarettes or have smoked over a period of six months and have been smoking over the past month; **Sex_Male**, male; **GoodSleep_Yes**, the participant felt he/she slept well and sufficiently.

**Reference**

Anderson, R.P., Jin, R., Grunkemeier, G.L., 2003. Understanding logistic regression analysis in clinical reports: an introduction. Ann. Thorac. Surg. 75 (3), 753–757. https://doi.org/10.1016/s0003-4975(02)04683-0.

Breiman, L., Friedman, J., Olshen, R.S., C., 1984. Classification and regression trees. Wadsworth International Group, Belmont, Calif.

Chen, T., He, T., Benesty, M., Khotilovich, V., Tang, Y., Cho, H., Chen, K., Mitchell, R., Cano, I., et al., 2023. xgboost: Extreme Gradient Boosting. https://CRAN.R-project.org/package=xgboost (accessed May 10 2024).

Choi, R.Y., Coyner, A.S., Kalpathy-Cramer, J., Chiang, M.F., Campbell, J.P., 2020. Introduction to Machine Learning, Neural Networks, and Deep Learning. Transl. Vis. Sci. Technol. 9 (2), 14. https://doi.org/10.1167/tvst.9.2.14.

Culp, M., Johnson, K., Michailidis, G., 2016. ada: The R Package Ada for Stochastic Boosting. https://CRAN.R-project.org/package=ada (accessed May 10 2024).

Friedman, J., Hastie, T., Tibshirani, R., 2010. Regularization paths for generalized linear models via coordinate descent. J. Stat. Softw. 33 (1), 1–22. https://doi.org/DOI 10.18637/jss.v033.i01.

Hofner, B., Mayr, A., Robinzonov, N., Schmid, M., 2014. Model-based boosting in R: a hands-on tutorial using the R package mboost. Computation. Stat. 29, 3–35. https://doi.org/10.1007/s00180-012-0382-5.

Karatzoglou, A., Smola, A., Hornik, K., Zeileis, A., 2004. kernlab - An S4 Package for Kernel Methods in R. J. Stat. Softw. 11, 1–20. https://doi.org/10.18637/jss.v011.i09.

Liland, K., Mevik, B., Wehrens, R., 2023. pls: Partial Least Squares and Principal Component Regression. https://CRAN.R-project.org/package=pls (accessed May 10 2024).

Venables, W.N., Ripley, B.D., Venables, W.N., 2002. Modern applied statistics with S, 4th ed. Springer, New York.

Wright, M.N., Ziegler, A., 2017. ranger: A Fast Implementation of Random Forests for High Dimensional Data in C++ and R. J. Stat. Softw. 77, 1–17. https://doi.org/10.18637/jss.v077.i01.
